# The gap between cause-of-death statistics and Household Registration reports in Shandong, China during 2011-2013: Evaluation and adjustment for underreporting in the mortality data for 262 subcounty level populations

**DOI:** 10.1371/journal.pone.0199133

**Published:** 2018-06-21

**Authors:** Jiandong Sun, Xiaolei Guo, Zilong Lu, Zhentao Fu, Xiujun Li, Jie Chu, Gaohui Zhang, Kou Kou, Fuzhong Xue, Aiqiang Xu

**Affiliations:** 1 School of Public Health and Social Work, Queensland University of Technology, Brisbane, Queensland, Australia; 2 Shandong Provincial Centre for Disease Control and Prevention, Jinan, Shandong, China; 3 Academy of Preventive Medicine, Shandong University, Jinan, Shandong, China; 4 School of Public Health, Shandong University, Jinan, China; Ball State University, UNITED STATES

## Abstract

Underreporting is a quality concern in mortality statistics. The purpose of this study was to assess and adjust underreporting in the population-based cause-of-death statistics. The total population (96 million) in Shandong, China was divided into 262 subcounty level populations geographically and by residence type (urban/rural). For each subpopulation, the total number of deaths during the years 2011–2013 was determined using data from the Household Registration System (HRS), and was used as a reference to assess the underreporting rate (UR) in the cause-of-death data from the Shandong Death Registration System (SDRS). It was estimated that 454,615 deaths, or 21.5% (95% CI: 21.4–21.5%) were unreported. Underreporting was more pronounced in rural (22.1%) versus urban communities (20.0%), in economically underdeveloped regions (32% versus 16% in least disadvantaged areas), and in newly included sites with no prior experience in cause-of-death reporting (24% versus 17%). Geographic variation was large with a UR at the prefectural level ranging from 11.2% to 43.7%. A stratified analysis showed that UR was higher in rural populations in high-income regions, but in middle- and low-income regions, was higher in urban communities. An adjustment factor (AF) was calculated for each of the 262 subpopulations (ranging from 0.9 to 2.5 with an average of 1.27). The total morality rate was adjusted from 6.03 to 7.67 deaths per 1000 persons. Underreporting in the SDRS varies greatly between areas and populations and is related to residence type, prior experience and local economy. Correcting underreporting at a local level is needed especially for comparative analyses across geographical areas or populations.

## Introduction

Underreporting or incompleteness is a common quality concern in the cause-of-death data from China [1–5]. At the national level, about 17% of deaths were uncounted in the data from the Disease Surveillance Points (DSP) system in 2006–2008, which included 161 points covering approximately 6% of the total Chinese population [6]. Another source for cause-of-death data is the Ministry of Health-Vital Registration (MOH-VR) system with a higher coverage (17% by 2012) [7]. The two systems were recently combined into an integrated national mortality surveillance system managed by the Chinese Centre for Disease Control and Prevention (CDC). This recently developed system is still a sample-based system covering about 24% of the total Chinese population from 605 county-level sampling sites [7].

A growing number of provinces, including Shandong, are extending their sample-based surveillance systems to their entire populations. Shandong is an eastern province with a population of 96 million in 2010 [8]. There were 142 county-level units (counties/cities/districts) at the end of 2013. Prior to 2010, reporting of cause-of-death data were routinely conducted in 30 counties or districts as part of the national systems (DSP and MOH-VR) and other projects. In 2010, cause-of-death reporting was introduced to all counties and districts and the “Shandong Death Registration System (SDRS)” was established. The SDRS covers the entire Shandong population. In 2013, 22% of the Shandong population was also included in the newly integrated national mortality surveillance system [7]. After the integration of the national system [7], the old systems including the DSP and the MOH-VR became a history at both national level and in Shandong Province.

Population-based mortality data were made available for the first time for this large and ethnically and culturally homogeneous population (99.3% are ethnically *Han* people). This represents a unique opportunity for examining “hot spots or clusters” of major causes of deaths, such as cancers. However, comparative analyses across geographic locations or subpopulations may be biased if data completeness or other quality issues differ between regions or populations. In Shandong, the overall level of underreporting in the former DSP sites (about 20 county-level units) was similar to the national results [6]. However, the great majority (80%) of sites are newly included with no prior experience. Even among the DSP sites, the inter-site variations in underreporting are unclear because of the relative small sample sizes in underreporting surveys.

Adjustment for underreporting is a common practice in mortality analysis and burden of disease studies [1–3, 9, 10]. The level of underreporting is often assessed by comparing the reported total number of deaths with a benchmark (representing the actual number of total deaths) which is usually acquired through surveys or statistical modelling, or from official government reports [3, 4, 6, 10–12]. However, this is usually done at the national or provincial level and there have been no recommendations or guidelines for dealing with this issue at a lower level, which limits the meaningful comparisons in mortality between smaller populations. The purpose of this study was to understand the level and nature of underreporting in the population-based cause-of-death data collected through the SDRS. This was performed by comparing the SDRS data with the official reports from the Household Registration System (HRS) at a subcounty level.

## Data and methods

Data were routinely collected through the population-based death registration system. No identifiable information was included in the data analysis. Results were all presented at aggregated levels. An ethics review was not required.

### Assessment units and urban-rural classification

The total Shandong population was firstly divided into 142 county-level populations based on administrative boundaries. Next, each of these 142 populations was further divided into two groups based on residential type: urban and rural. The rural population was defined as people living in rural townships. A township is an administrative unit consisting of a town centre and dozens of surrounding villages. The urban population was defined as those who reside in subdistricts (urban suburbs), which are made up of urban communities or neighbourhoods clustering around the centre of a county/city/district.

For 17 districts, there are not any townships and therefore the total population was defined as urban only. SDRS death data from 5 counties or cities lacked classifiable residential information due to different data format. Most residents in these locations live in townships and we defined the whole populations as rural. Finally, the total Shandong population was divided into 262 subcounty-level populations. Among them, 137 were classified as urban and 125 as rural populations. The population size (in 2010) ranged from about 21,000 to 1,365,000 with an average of 367,000 persons.

One-fourth (n = 66) of these subpopulations were previously involved in cause-of-death reporting prior to 2010. Based on the 2013 per capita disposable income (for urban populations) and the 2013 per capita net income (for rural populations) [13], a three-level (low, middle and high) income variable was created with a similar number of units in each category.

### SDRS data

As mentioned earlier, the SDRS commenced in 2010 to collect information on all deaths in the Shandong population. It is managed by the Shandong Centre for Disease Control and Prevention (CDC). Reporting is undertaken primarily within the health system with support from police departments, which manage the HRS and coronial investigation, and bureaus of civil affairs which regulate cremation practice. Data are collected by local hospitals and CDCs using a standard protocol previously developed for the DSP system [2]. Specifically, for deaths occurred in hospitals, a death certificate is issued by the doctor in-charge. For deaths occurred at home and other places, a death certificate is completed by village doctors or local hospitals based on medical history and/or an interview with family members. For deaths requiring coroner investigation, a coroner report is used. It is required by government regulations that the family should present these certificates to police departments for HRS deregistration and to bureaus of civil affairs for cremation. At the same time, local hospitals and local CDCs collect these records and entre the data into the SDRS via the online direct-reporting platform. For deaths occurred in the internal migrants who live away from their registered residence, HRS deregistration needs to be done in the police departments in their original residence. Theoretically, these deaths are included in both the HRS data and the SDRS data, although there might be reporting delays compared to local reporting.

There are mainly three possible reasons for unreporting. Firstly, some families may deliberately avoid HRS deregistration or cremation by not going through the official procedures. However, this is deemed unlawful and is reasonably rare. Secondly, because the protocol was new, in some regions HRS deregistration and cremation may be undertaken without checking death certificates. Such deaths will be recorded in the HRS and cremation lists, but usually not in the SDRS. Based on our experience, this is the most important reason for underreporting. Thirdly, underreporting may happen due to data management errors in the reporting chain, such as failing to enter data into the system for deaths with a certificate.

In 2010, reporting did not commence in a small number of counties due to personnel and logistic challenges; but all started data collection by 2011. Therefore, we used 2011–2013 data for this analysis. We extracted all deaths reported to the SDRS (Oracle database) during the years 2011–2013. Variables included death ID, sex, age, residence and underlying cause of death. A total of 1,682,048 deaths were initially included. Among them, 496211 (29.5%), 576904 (34.3%) and 608933 (36.2%) were reported in 2011, 2012 and 2013, respectively. A check for reporters suggested that in 14 counties in 2011, and in 1 county in 2012, reporting did not cover all townships or subdistricts. The death records from these counties during these years (n = 15,018, 0.9% of all reported deaths) were excluded. Further, 839 deaths (0.05%) were excluded due to invalid coding in residential address. Finally, all <1-year deaths (n = 8785, 0.5%) were removed, resulting in 1,657,406 deaths included in this analysis. We did not include infant deaths because very few of them are actually captured by the HRS. All years’ data were pooled and a total number of SDRS-deaths (≥1 year) was calculated for each of the 262 subpopulations.

### HRS data

In China, population movement, including birth, death and migration is closely monitored by local police departments according to the Regulations of the People’s Republic of China for Household Registration [14, 15]. When a death occurs, the family members have the responsibility to report to the local police department for deregistration [16]. There is also a government regulation to have the body cremated in most areas. Anecdotally, some deaths may be intentionally unreported to avoid cremation for cultural reasons in some ethnic regions. This is unlikely to have a significant impact on the HRS data in Shandong because 99.2% of the Shandong population are ethnically *Han* Chinese and are highly homogeneous in culture [13]. Mortality data from censuses are considered to be most complete [17]. In fact, the total death rates in Shandong based on HRS data are often similar or even higher than the census data [13], suggesting good completeness.

Among other indicators, the yearly numbers of deaths based on the HRS data are routinely reported at a township or subdistrict level internally within government departments. Such data are aggregated to higher levels for public reports, such as the statistic year books. It should be noted, however, that these data are organised based on the date of deregistration rather than the date of death. Because of the reporting lag time, for deaths occurred at the end of year may be reported in the following year or even later, which may result in some unexpected yearly variations and a certain level of inconsistency between the HRS and the SDRS. To minimise this effect, we prepared the average death rates for a period of 3 consecutive years using HRS data.

At the outset of this study (early 2014), HRS reports for 2013 was not available. We collected all annual reports with township/subdistrict breakdowns for the years 2010–2012 from official publications (statistical year-books) or directly from local police departments or statistical bureaus. We aggregated these data for the 262 subpopulations based on aforementioned classification method. The average death rate in 2010–2012 was calculated for each subpopulation and was used as a proxy for the rate for 2011–2013. Next, we applied these rates to the 2011–2013 populations to estimate the total numbers of deaths for all subpopulations for years which were consistent with the SDRS reporting. For the counties/districts (n = 15) with only 2012–2013 or 2013 data, we used HRS data from the same period to ensure comparability.

### Statistical analysis

Data were prepared and analysed using the R Software [18]. We considered the difference between the number of HRS-deaths (D_HRS_) and the number of SDRS-deaths (D_SDRS_) to be unreported deaths in the SDRS, and thus defined the UR = (DHRS−D_SDRS_) / D_HRS_ × 100%. Wald 95% confidence intervals (CIs) based on binomial distribution were calculated. Geographic variation was presented at the prefectural level. A multiple logistic regression analysis was performed to assess the independent effects (represented as odds ratios [ORs] and 95% CIs) of residence type (urban/rural), income level and prior experience on underreporting controlling for regional variations.

An adjustment factor (AF) was calculated as the ratio of D_HRS_ / D_SDRS_ for all 262 subpopulations. These AFs were applied to the unit record data from the SDRS to generate underreporting-corrected mortality estimates at the subcounty level. For example, an AF of 1.2 means each reported death is counted as 1.2 deaths in the adjusted analysis.

## Results

### Underreporting rates

During the years 2011–2013, the total number of deaths (≥1 year) reported to the SDRS was 1,657,406, and the total number of deaths based on HRS data was 2,112,021. It was estimated that 454,615 deaths were unreported in this CDC-based cause-of-death reporting system, resulting in an overall UR of 21.5% (95% CI: 21.4–21.5%).

**Fig 1:**
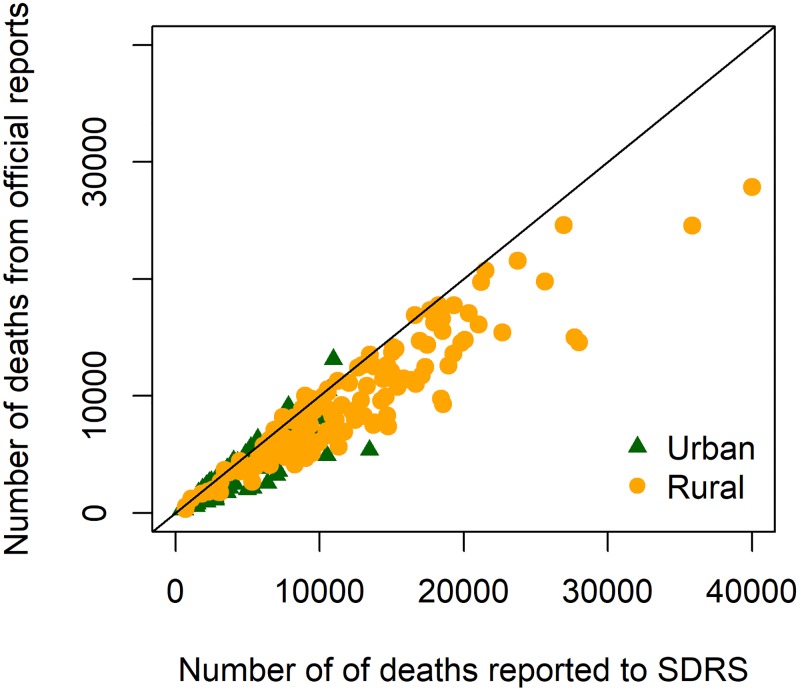
Numbers of deaths reported to the SDRS and numbers of deaths from the HRS for 262 subpopulations in Shandong, 2011–2013. HRS = Household Registration System; HDRS = Shandong Death registration System.

The total number of deaths (≥1 years) for each of the 262 subpopulations ranged from 246 to 27842 in the SDRS, and 382 to 40004 in the HRS. The distribution of these data points stratified by rural/urban residence is presented in Fig 1. The two sets of data were highly correlated (r = 0.95). As expected, the number of deaths in the SDRS was smaller than the number of deaths in the HRS for the great majority of the assessment units. However, for a small number of units (n = 28, 11%), the number of deaths was higher in the SDRS (above the diagonal line, Fig 1). When aggregated to the county level, slightly more deaths from the SDRS were observed in 9 (6%) out of the 142 county-level units.

As shown in Table 1, UR was higher in rural versus urban areas (22.1% vs. 20.0%), and in newly included counties or districts with no prior experience (23.5% vs. 16.9%). The UR was much higher among low-income regions (32.3%) compared to high- and middle-income regions (15.6% and 18.8%, respectively). Geographic variation at the prefectural level was large with an UR ranging from 11.2% to 43.7%.

**Table 1:** Underreporting rates in the cause-of-death data from the Shandong Death Registration System (SDRS), China, 2011–2013.

	Deaths in HRS	Death in SDRS	UR (95%CI)
Total	2112021	1657406	21.5 (21.5–21.6)
Residence type			
Urban	593269	474885	20.0 (19.9–20.1)
Rural	1518752	1182521	22.1 (22.1–22.2)
Income level			
High	679812	573833	15.6 (15.5–15.7)
Middle	842617	684327	18.8 (18.7–18.9)
Low	589591	399246	32.3 (32.2–32.4)
Prior experience			
Yes	635539	528232	16.9 (16.8–17.0)
None	1476482	1129174	23.5 (23.5–23.6)
Prefecture			
Binzhou	98025	63215	35.5 (35.2–35.8)
Dezhou	138864	98499	29.1 (28.8–29.3)
Dongying	27778	23041	17.1 (16.6–17.5)
Heze	192313	108206	43.7 (43.5–44.0)
Jinan	138025	115900	16.0 (15.8–16.2)
Jining	173859	127046	26.9 (26.7–27.1)
Laiwu	27426	24165	11.9 (11.5–12.3)
Liaocheng	119210	101087	15.2 (15.0–15.4)
Linyi	224374	189242	15.7 (15.5–15.8)
Qingdao	181658	154644	14.9 (14.7–15.0)
Rizhao	51485	29948	41.8 (41.4–42.3)
Tai’an	129832	112809	13.1 (12.9–13.3)
Weifang	189101	167850	11.2 (11.1–11.4)
Weihai	63520	53445	15.9 (15.6–16.1)
Yantai	175163	147286	15.9 (15.7–16.1)
Zaozhuang	87720	61506	29.9 (29.6–30.2)
Zibo	93668	79517	15.1 (14.9–15.3)

Notes: HRS = Household Registration System; HDRS = Shandong Death registration System; UR = underreporting rate

**Fig 2:**
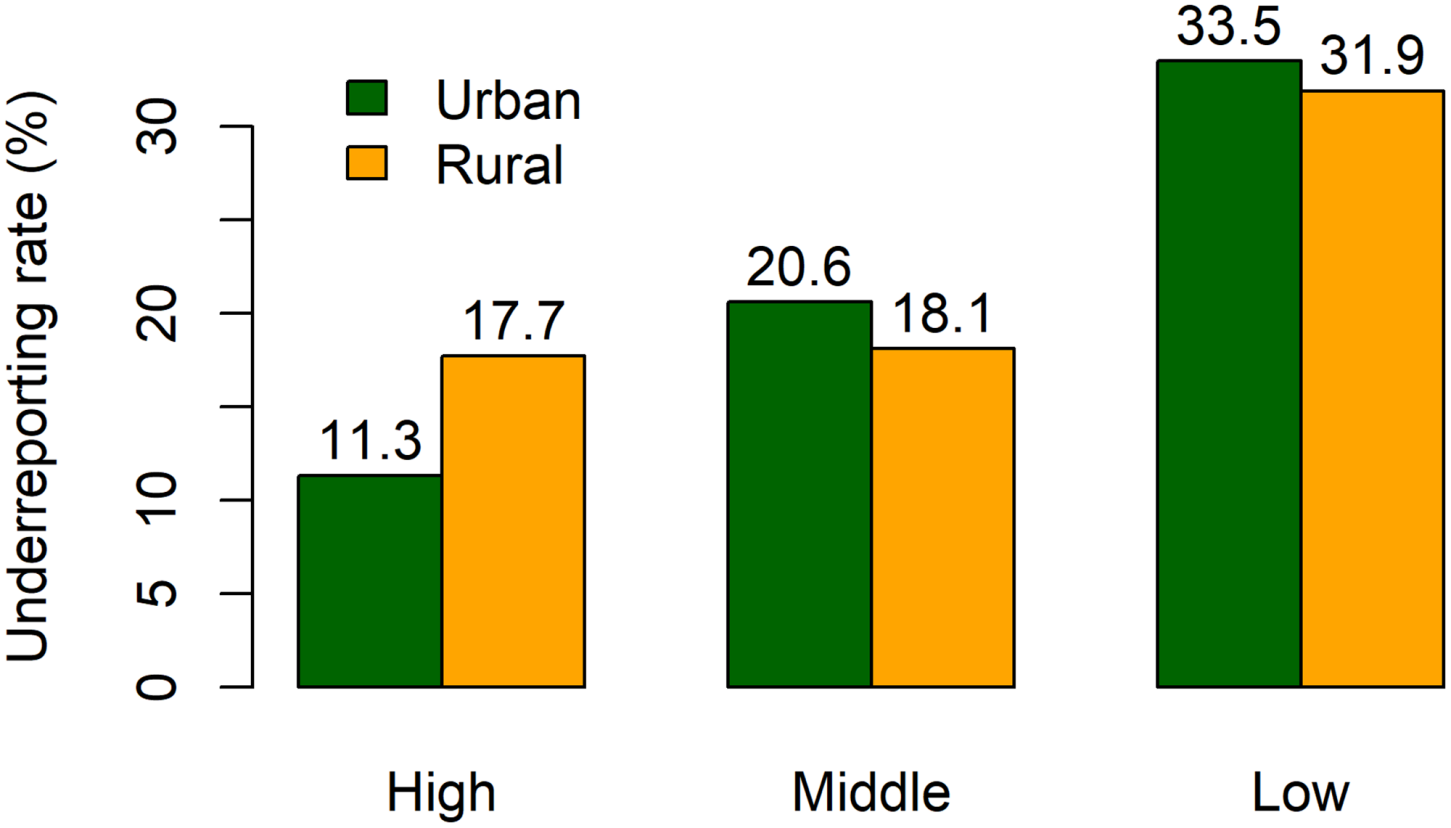
Underreporting rates by residence type (urban/rural) and economic level in the cause-of-death data from the Shandong Death Registration System, 2011–2013.

A multiple logistic regression analysis showed underreporting was significantly (95%CI for OR not including 1) more common in urban vs. rural areas (OR = 1.19, 95%CI: 1.18–1.20), in areas without prior experience (1.72, 1.70–1.74), and in middle- (2.78, 2.73–2.84) and low-income (4.96, 4.88–5.05) vs. high-income regions. Surprisingly, urban residence was found to be associated with more underreporting in this model. A stratified analysis showed that, in high-income regions, UR was much higher in rural areas; while in middle- or low-income regions, urban population had a higher UR (Fig 2).

### Adjustment for underreporting

An adjustment factor (AF) was calculated for each of the 262 subpopulations. It ranged from 0.9 to 2.5 with a provincial mean of 1.27. The total morality rate per 1000 persons was adjusted from 6.03 to the level consistent with the HRS data (7.67 deaths). These AFs were also used to adjust cause-specific rates at the subcounty level. Table 2 shows the uncorrected and underreporting-corrected mortality rates for lung cancer as an example. This adjustment did not change the distributions by gender, age or cause of death, or relative contributions of a cause among all causes, but the absolute values of mortality rate and in turn the ranks of specific cause of death across subpopulations. For example, the mortality rate in Heze nearly doubled with a rank jumped from 15^th^ to 7^th^ after adjustment.

**Table 2:** Uncorrected and underreporting-corrected mortality rates for lung cancer in Shandong, China, 2011–2013.

	Uncorrected			Corrected for underreporting		
	% of total deaths	ASR	Ranking	% of total deaths	ASR	Ranking
Dongying	11.0	54.42	1	11.0	65.21	1
Laiwu	8.9	51.00	2	8.9	59.06	2
Weifang	9.0	46.64	3	9.0	53.13	5
Zibo	8.9	44.27	4	8.9	52.46	6
Tai’an	7.7	43.89	5	7.7	50.52	8
Qingdao	8.4	41.43	6	8.4	50.29	9
Zaozhuang	8.1	39.08	7	8.1	56.44	4
Yantai	7.3	38.27	8	7.3	45.99	10
Jinan	6.9	36.98	9	6.9	44.19	12
Binzhou	7.6	36.65	10	7.6	56.80	3
Linyi	6.8	35.73	11	6.8	43.22	14
Weihai	7.8	34.84	12	7.8	43.55	13
Liaocheng	5.9	30.58	13	5.9	36.61	17
Jining	6.1	28.06	14	6.1	40.38	15
Heze	6.5	29.97	15	6.5	52.31	7
Dezhou	5.3	27.55	16	5.3	40.34	16
Rizhao	7.4	25.34	17	7.4	44.70	11
Shandong	7.3	37.02			47.64	

ASR = age-standardised rate (deaths per 100,000 persons) to the 2010 Chinese census population

## Discussion

Mortality assessments at a local level will help researchers and policy-makers to identify inequalities in leading causes of deaths and areas or subpopulations with elevated risks. This requires that data quality issues including incompleteness to be addressed at a local level. To the best of our knowledge, this is the first study to systematically evaluate underreporting in mortality data from China at a lower-then-provincial level. We estimated the degree of underreporting in the data from the Shandong Death Registration System (SDRS) for 262 subpopulations by comparing these data with the independently acquired official estimates from the Household Reregistration System (HRS). The estimated URs were subsequently used to correct incompleteness in the site- and cause-specific mortality rates.

The reference data is the key for assessing underreporting that are usually acquired through surveys, statistical models or official reports [3, 4, 6, 10–12]. If available, official data are likely to be a cost-effective source for this purpose. Using a similar technique, Phillips and colleagues [10] adjusted the number of suicide deaths in China during 1995–99 based on the gap between the number of all deaths in the Ministry of Health-Vital Registration (MOH-VR) system and the expected number of deaths based on the 1% sample survey data by the Bureaus of Statistics. The reported suicide rate in the MOH-VR was adjusted upward by 18% to correct the underestimation due to unreporting [10].

In China, there are mainly three official sources for total mortality: census data, 1% sampling survey data, and the HRS summary data. Census data are available only for census years; the other two are reported annually. Survey data at a provincial level are publicly available and thus can be used to adjust mortality rates at a provincial or higher level [10]. However, our aim was to assess URs for all populations at a sub-county level and therefore the survey data are no longer adequate. The HRS reports are available at a subdistrict/township level and therefore were chosen as our reference data.

The completeness of our reference data was also indirectly supported by our recent underreporting survey. It was conducted in early 2014 with a total sample of about 1 million people from 42 randomly selected township-level sites [19]. The purpose of this survey was to obtain high-level estimates of underreporting, such as the underreporting rates by gender, age groups, broad cause-of-death category, and level of reported death rates. However, these data were not sufficient to adjust death rates at a county/district level. The overall UR in the 2012–13 SDRS mortality data was estimated to be 23.2% for all ages, and 22.3% (calculated based on raw data) for deaths ≥ 1 year. The latter was similar to the overall UR estimate in the current analysis (21.5%, 95%CI: 21.4–21.5%).

The overall UR in Shandong seems much higher than the national estimate (16.7% in 2006–08) [6]. However, they are not directly comparable because the national result is from the DSP sites. Our cause-of-death reporting covers the entire population and the majority sites are newly included after 2010. In our subpopulations with prior experience (most of them were former DSP and MOH-VR sites), the average UR is 16.9 (95%CI: 16.8–17.0), which is almost identical to the national estimate. The high consistency with survey-based estimates at either provincial or national level may suggest good validity and feasibility in using HRS data for assessing underreporting.

The method used in this study also has the potential to overcome some mismatch between the death and population data. The SDRS is essentially a reporting system for deaths. Population data are collected separately usually at the beginning of the following year. There is a risk of mismatch especially when there are changes of catchment boundaries. This was found to be the main reason for the higher numbers of SDRS-deaths in some of our subpopulations (n = 28). In contrast, the deaths and population in the HRS data are always from the same cohort. By adjusting to the HRS level, we could correct the possible overestimation or underestimation due to this possible mismatch in the SDRS.

It should be noted that due to data availability, we used 2010–2012 HRS rates as the reference to estimate the underreporting rates in the 2011–2013 SDRS data. Essentially, we have adjusted the mortality rates to the level of 2010–2012 HRS rates. While acknowledging that reference data from exactly the same period should be used when possible, we argue that this practice shouldn’t result in a marked difference because the yearly variation in the total death rate was small for large populations and two of the three years overlap. More importantly, the primary focus in our work is to remove the potential differential effects of underreporting across regions and population groups. Therefore, the key to this method is to find a set of reference data that are comparable between subpopulations.

There are several issues related to the HRS data. First, active collection is required because most of such data are not publicly available. Good support from local Police Department or Bureau of Statistics is critical to collect such data when they are not published. Second, most infant (<1 year) deaths are not included in the HRS data because they are not yet registered to the system. Over half (50%) of these deaths occur in the first week and about 70% in the first month of life [20], while the recommended time for birth registration is 1 month [16]. Therefore, we only assessed underreporting for non-infant deaths. Underreporting of infant deaths needs to be assessed and adjusted separately. Third, the numbers of deaths in the HRS reports are by definition numbers of de-registrations. Delays or changes in reporting might cause some year-to-year deviations between the number of de-registrations and the actual number of deaths. Although we pooled three consecutive years’ data to smooth out the potential variation, there might still be slight inconsistencies between the two data sources. Accordingly, we did not assess the year-specific underreporting rates in the SDRS data.

The exclusion of 839 deaths due to invalid coding in residential address may have potentially caused a slight overestimation of the underreporting rates. However, this number was very small (0.05% of all deaths) and therefore its effect is likely to be negligible in practice.

As expected, we found underreporting is much more common in areas that are economically disadvantaged and in the newly included sites without prior experience. The performance of local CDCs is generally poorer in economically underdeveloped regions compared to more affluent areas. It is also true for other government agencies, such as the police and statistics departments. This could help explain the observed relationship. More attention need be paid to these underdeveloped sites for quality control and improvement in death reporting. The difference in UR between experienced and new sites suggests that data completeness will increase over time when new sites acquire more experience. In fact, our recent survey also showed a significant reduction in the overall UR from 27% in 2012 to 20% in 2013 [19].

Slightly more underreporting is observed in rural versus urban areas in our crude analysis (22% vs 20%) which is consistent with results from periodic surveys [1, 2, 6]. However, the direction of association reversed in our multiple analysis, with urban areas having a higher risk of underreporting. By stratified analysis (Fig 2), we found that underreporting is more common in rural communities in developed regions, while is higher in urban communities in middle- and low-income regions. The exact reasons for this finding need to be further investigated. This may be related to health infrastructure and internal migration. In China, internal migration from rural to urban areas is placing enormous pressure on the health system and infrastructure [21]. Many local CDCs in middle-and-low-income regions are struggling to fulfil public health duties for their urban communities mainly due to the huge gap between the existing health infrastructure including reporting capacity and the ever-growing city population and area. On the other hand, public health network including local hospitals are already developed in the districts of large cities (which are usually more developed in economy) and the impact of internal migration is less pronounced.

Variations in underreporting across regions and populations suggests a great need for correction of incompleteness. As shown in Table 2, the mortality burden based on uncorrected data will be substantially underestimated in areas with poor reporting quality. Data incompleteness should always be assessed and corrected when appropriate before any comparative analyses, such as spatial clustering analysis, are performed.

Due to lacking of information in the HRS data, we were unable to examine if underreporting is associated with gender, age and cause of death. However, as mentioned earlier, the majority of unreported cases in the SDRS are already included in the HRS and the missing is mainly due to poor communication between hospitals and CDCs with police departments who are responsible for checking death certificate before HRS deregistration. Theoretically, unreporting in SDRS is unlikely to differ based on individual characteristics of the diseased and their families. There is also supporting evidence from our recent survey which showed no differences in the UR between genders and across broad groups of causes of death [19]. Previous studies have shown underreporting is more common in infant and child deaths [2, 6, 19]. However, as mentioned earlier, our URs are used to correct non-infant deaths only. Moreover, deaths at a young age (<15 years) are rare, accounting for only 1% of the total deaths. Despite this age effect, our URs should be adequate to correct the cause-specific mortality for most major causes (such as cancers) that are rarely occurred in children and young people.

To conclude, underreporting in the population-based cause-of-death data in Shandong is high. Slightly more than one-fifth of the total deaths are unreported. Underreporting differs substantially across regions and populations and is related to lack of experience and economic underdevelopment. Comparisons between regions or subpopulations based on uncorrected data could be heavily biased. The HRS data appear to be a good reference for assessing underreporting at an aggregated community (sub-county) level.

## Supporting information

S1 Dataset (XLSX)

## References

[1] Rao C, Lopez AD, Yang G, Begg S, Ma J. Evaluating national cause-of-death statistics: Principles and application to the case of China. Bulletin of the World Health Organization. 2005;83(8):618–25. 16184281 PMC2626325

[2] Yang G, Hu J, Rao KQ, Ma J, Rao C, Lopez AD. Mortality registration and surveillance in China: History, current situation and challenges. Population Health Metrics. 2005;3(3):1–9. 15769298 10.1186/1478-7954-3-3 PMC555951

[3] Lopez AD, Salomon J, Ahmad O, Murray CJ, Mafat D. Life tables for 191 countries: Data, methods and results. GPE Discussion Paper Series: No9 [Internet]. 2000 30 June 2016:[6–7 pp.]. http://www.who.int/healthinfo/paper09.pdf.

[4] Li Q, Zhou M, Bishai D, Wang L, Ma S, Hyder A. The under-report adjustment of injury deaths data from National disease surveillance points system of China. Injury Prevention. 2012;18(Suppl 1):A232.

[5] Banister J, Preston SH. Mortality in China. Population and Development Review. 1981;7(1):98–110.

[6] Wang L, Wang L-j, Cai Y, Ma L-m, Zhou M-g. 2006–2008年全国疾病监测系统死亡漏报调查分析 [Analysis of under-reporting of mortality surveillance from 2006 to 2008 in China]. Zhonghua Yu Fang Yi Xue Za Zhi. 2011;45(12):1061–4. 22336336

[7] Liu SW, Wu XL, Lopez AD, Wang LJ, Cai Y, Page A, et al An integrated national mortality surveillance system for death registration and mortality surveillance, China. BULLETIN OF THE WORLD HEALTH ORGANIZATION. 2016;94(1):46–57. doi: 10.2471/BLT.15.153148 26769996 10.2471/BLT.15.153148 PMC4709796

[8] Shandong Office for the 6th Census. Shandong primary data report of the 2010 6th Census 2011 [Dec 12, 2012]. http://www.stats-sd.gov.cn/disp/tjgb.asp?aa=4220100001.

[9] Zhou M, Wang H, Zhu J, Chen W, Wang L, Liu S, et al Cause-specific mortality for 240 causes in China during 1990–2013: a systematic subnational analysis for the Global Burden of Disease Study 2013. Lancet (London, England). 2016;387(10015):251–72. 10.1016/S0140-6736(15)00551-6 26510778

[10] Phillips MR, Li X, Zhang Y. Suicide rates in China, 1995–99. The Lancet. 2002;359(9309):835–40. 10.1016/S0140-6736(02)07954-0 11897283

[11] Wan X, Zhou M-g, Wang L-j, Chen A-p, Yang G-h. 运用广义增长平衡法和综合绝世后代法估计1991–1998年全国疾病监测系统的居民漏报水平 [Using general growth balance method and synthetic extinct generations methods to evaluate the underreporting of death at disease surveillance points from 1991 to 1998]. Zhonghua Liu Xing Bing Xue Za Zhi. 2009;30(9):927–32. 20193230

[12] Lo S-H. Estimating a survival function with incomplete cause-of-death data. Journal of Multivariate Analysis. 1991;39(2):217–35.

[13] Shandong Provincial Bureau of Statistics. Shandong Statistical Yearbook—20142014 11 July 2016. http://www.stats-sd.gov.cn/tjsj/nj2007/indexch.htm.

[14] Cui R, Cohen JH. Reform and the HuKou System in China. Migration Letters. 2015;12(3):327.

[15] Chan KW. The Chinese Hukou System at 50. Eurasian Geography and Economics. 2009;50(2):197–221.

[16] 全国人民代表大会 [The National People’s Congress of the People’s Republic of China]. 中华人民共和国户口登记条例 [Regulations of the People`s Republic of China on Household Registration]1 July 2016. http://www.npc.gov.cn/wxzl/gongbao/2000-12/10/content_5004332.htm.

[17] Banister J, Hill K. Mortality in China 1964–2000. Population Studies. 2004;58(1):55–75. doi: 10.1080/0032472032000183753 15204262 10.1080/0032472032000183753

[18] Team RDC. R: A language and environment for statistical computing. Vienna, Austria: R Foundation for Statistical Computing; 2010.

[19] Zhang G, Guo X, Lu Z, Sun J, Xu A. Estimation of the underreporting rate of resident death by capture-mark-recapture method in Shandong Province. Chinese Journal of Prevention and Control of Chronic Diseases 2015;23(5):325–7.

[20] Wang Y, Zhu J, He C, Li X, Miao L, Liang J. Geographical disparities of infant mortality in rural China. Archives of Disease in Childhood Fetal and Neonatal Edition. 2012;97(4):F285–F90. doi: 10.1136/archdischild-2011-300412 22247413 10.1136/archdischild-2011-300412 PMC3391502

[21] Hu X, Cook S, Salazar MA. Internal migration and health in China. The Lancet. 2008;372(9651):1717–9. 10.1016/S0140-6736(08)61360-4 PMC7135200 18930533

